# Universal inherent fluctuations in statistical counting of large particles in slurry used for semiconductor manufacturing

**DOI:** 10.1038/s41598-020-71768-3

**Published:** 2020-09-07

**Authors:** Manhee Lee, Dongwon Kim, Tae-Young Heo, Taewon Park, Wonjung Kim, Daejin Choi, Hyunwoo Kim, Jaehyun Kim

**Affiliations:** 1 Department of Physics, Chungbuk National University, Cheongju, Chungbuk 28644 Korea; 2 Department of Information and Statistics, Chungbuk National University, Cheongju, Chungbuk 28644 Korea; 3 Samsung SDI, Gongse-ro, Giheung-gu, Yongin-si, Gyeonggi-do 17084 Korea; 4 Samsung Electronics, Kiheung Campus, Yongin-si, Gyeonggi-do 446-811 Korea; 5 Present Address: School of Economics and Management, Tsinghua University, Beijing, China

**Keywords:** Nanoparticles, Statistical physics, Characterization and analytical techniques, Chemical engineering, Colloids

## Abstract

In the chemical mechanical polishing process of semiconductor manufacturing, the concentration of ‘large’ particles ($\ge$0.5 μm) in the slurry, which is considerably larger in size than the main abrasives ($\approx$ 0.1 μm), is a critical parameter that strongly influences manufacturing defects, yields, and reliabilities of large-scale-integrated circuits. Various instruments, so-called particle counters, based on light scattering, light extinction, and holography techniques have been developed to measure and monitor the large particle concentration in semiconductor fabs in real time. However, sizeable fluctuation in the measured particle concentration complicates the statistical process control in the fabs worldwide. Here, we show that an inherent fluctuation exists in the counting of large particles, which is universal, independent of instrument type, and quantitatively determined by the instrument’s operation parameters. We analytically derive a statistical theory of the fluctuation based on Poisson statistics and validate the theory through experiments and Monte-Carlo simulation. Furthermore, we provide a strategy to enhance the measurement accuracy by statistically adjusting the instrumental parameters commonly involved in the particle counters. The present results and analyses could be useful for statistical process control in semiconductor fabs to prevent large particle-induced defects such as micro-scratches and pits on wafers.

## Introduction

Recent semiconductor manufacturing shows that the quality of materials used in each process strongly influences manufacturing defects, yields, and reliabilities of the final products. Although numerous material parameters determine material quality, a common critical parameter is the concentration of undesirable particulate matter in materials^1^. As the particulates act as sources of severe manufacturing defects such as micro-scratches^2^, pits^3^, and voids^4^, the concentration of nanoscale to microscale particulates should be accurately measured, controlled, and monitored to prevent such material-induced defects^5^. Various types of particle-sizing and counting instruments have been developed and used, based on light scattering^6,7^, light extinction^8–10^, and hologram techniques^11–13^, which commonly measure the minute volume of liquid samples on the micro- to milli-liter scales.

For slurry solutions, which are used for chemical mechanical polishing (CMP), a Large Particle Counter (LPC) is used to measure “large” particles that are much larger in size than the main abrasives (~ 0.1 μm)^14^. In our study, we define the large particle as the particles with diameter greater than 0.5 μm. Owing to the limited optical resolution of the LPC, the turbid slurry sample is first diluted a few ten times using deionized water. Moreover, the diluted slurry flows into the detection module of the LPC at a specific flow rate, about tens of milliliters per minute, wherein the number of large particles is counted for a given period, typically a few minutes. Finally, the concentration of large particles, i.e., the particle number per 1 *ml*, is calculated from the measured experimental data. Consequently, the net volume of the slurry used is typically about tens of microliters for a single measurement.

Although the experimental and instrumental details including dilution ratio, flow rate, and optical detection are precisely made in the LPC or relevant techniques, an intrinsic Poisson fluctuation always exists in the number of particles counted^15^. In Poisson statistics, because the number variation relative to the average number is inversely proportional to the average number, the measurement of samples having minute volumes severely deteriorates statistical accuracy, increasing the relative error. To achieve a desired accuracy, one should statistically adjust the diluent ratio, measurement volume, and the number of measurements, in addition to the precisely controlled experimental details.

Here, we analytically derive the probability density function for the number of large particles measured via LPC, taking into account the measurement parameters that are commonly involved in LPC. We show that the intrinsic relative error or the fluctuation, defined by the half width of 95 % confidence interval of the expected average number divided by the average number itself, is inversely proportional to the square root of the average number of large particles in a given volume. Therefore, for a given dilution factor associated with the technical limitation of optical detection, the number of measurements or measurement volume could be statistically adjusted to obtain the concentration of large particles with a desired accuracy.

**Figure 1:**
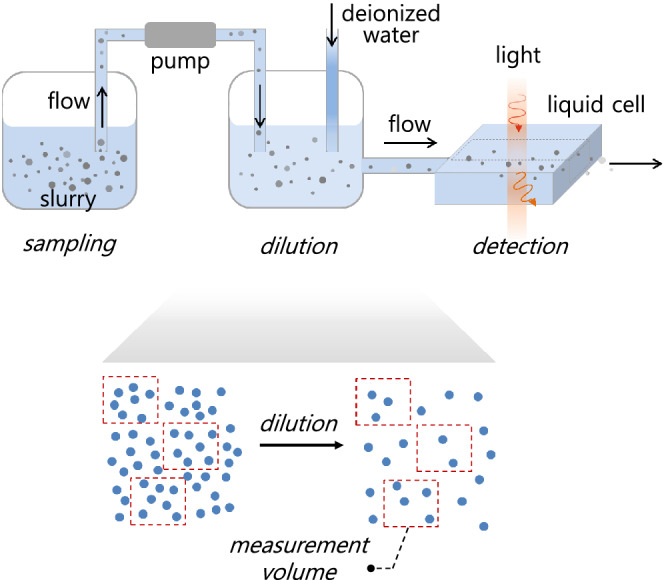
Experimental procedure for measuring “large” particle concentration in a slurry. First, a silica slurry solution is extracted into the measurement equipment through an installed syringe pump. Second, the slurry is diluted with deionized water, such that the particle concentration decreases to less than the maximum detectable concentration (9000 ea/ml for the equipment used herein). Finally, a light scattering technique is used to measure the size of individual particles flowing through the detection channel, from which the particle number by size is obtained. By considering the dilution factor and the measured volume of the diluted solution, the number of particles per 1 ml is calculated. We have used a commercial “Particle Counter (AccuSizer 780 APS, Particle Sizing Systems)” and a commercial slurry of silica particles.

## Experiment of “Large” particle counts in slurry

Large particles in a slurry, either formed by particle aggregation or brought by contamination, induce severe manufacturing defects such as micro-scratches^16^ and pits^17^, and thus the particle concentration is measured and monitored in real time during semiconductor manufacturing through LPC. Although various types of LPCs^6,8,11^ are available, they mostly follow the experimental procedure described in Fig. 1. The slurry is first diluted using deionized water to reduce the particle concentration to be relevant to optical detection resolution, and then a specific volume of diluted slurry is introduced into the optical detection module to measure the size of the particles and count their numbers therein. Finally, the instrument provides the particle concentration, the number of particles per ml, calculated from the measured counts. This procedure is repeated several times to obtain the mean value of the particle concentration. Here, we notice that relative fluctuation, fluctuation divided by mean, of the diluted sample is relatively higher than that of the original concentrated sample (Fig. 1, bottom).

**Figure 2:**
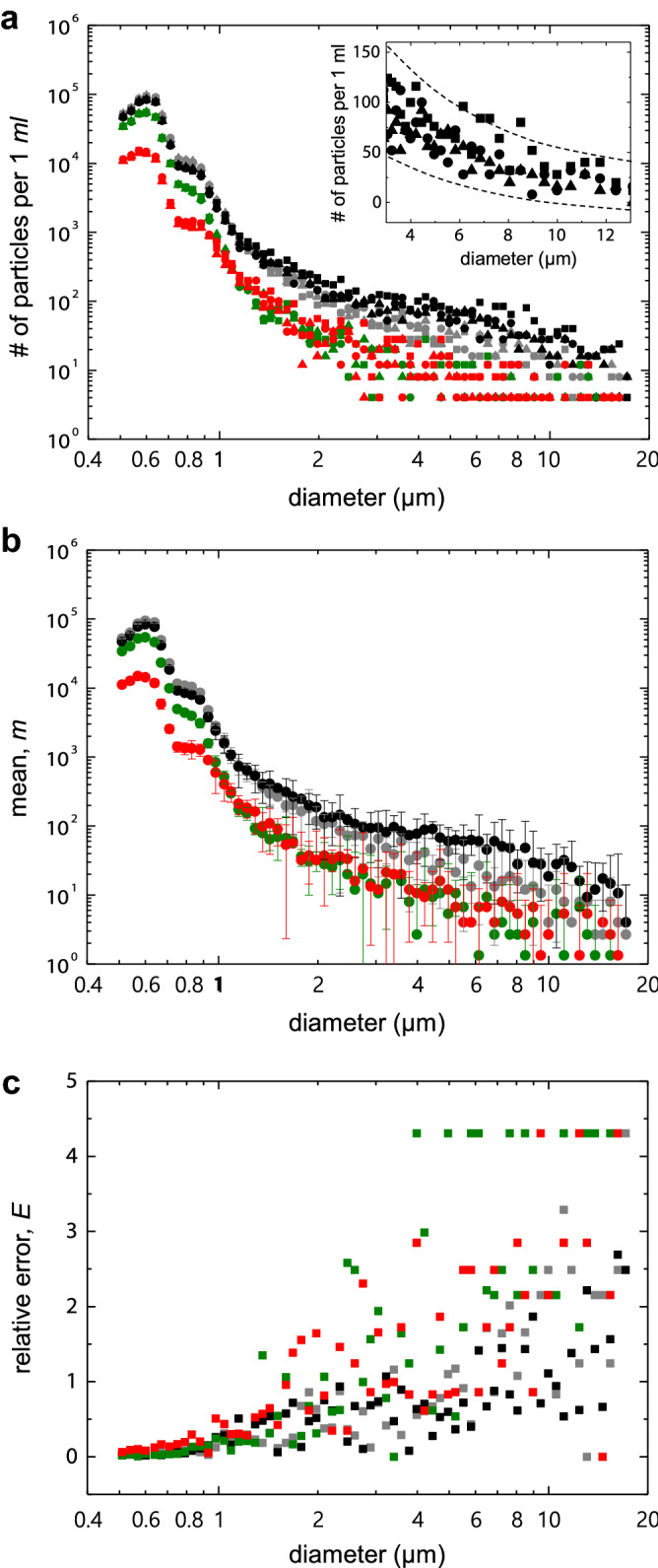
Experimentally obtained particle concentrations for four silica slurries with different size distributions. (**a**) We used one freshly prepared slurry (black symbols), two filtered slurries of the original one through two different sized pores (green and red symbols), and the original slurry after three weeks of aging in a bottle at ambient condition (grey symbols); we performed three successive measurements for each slurry (rectangles, triangles, circles). Inset: a closer look at the particle concentration of the original fresh sample (black symbols) in linear scales at a particle diameter ranging between 3 and 9 μm. (**b**) The mean values with 95 % confidence interval of the three successive measurements of the four slurries shown in (**a**). (**c**) The relative error *E*, defined by Eq. (1), for the four slurries. In the experiments, a dilution factor of $\alpha$=60 was used, where $\alpha$ is defined as the total volume of the mixed solution of the slurry and deionized water divided by the pure slurry volume. The measured volume of the diluted slurry is $\beta \,ml$; here, the volume factor $\beta$=15.

Figure 2a shows the particle concentration measured by a commercial LPC (Accusizer 780 APS, Particle Sizing Systems), which follows the procedure described in Fig. 1 and provides the concentration of particles larger than 0.5 μm. We have used four slurries of silica colloids: an original fresh slurry (black symbols), two slurries filtered through two depth filters of different sized pores (green and red symbols), and the original slurry after 3 weeks of aging in a bottle at ambient condition (grey symbols). We have performed three consecutive measurements for each slurry (rectangles, triangles, and circles). While the three measurements are continuously made through the instrument used, fully stirring between each measurement would suppress unintended changes in the solution condition, such as the increase of large particles by the aggregation of small particles. As expected, the two filtered slurries (green and red symbols) have smaller size distributions than the original fresh one (black symbols). We obtained a smaller number of particles with smaller pore filters (green $\rightarrow$ red symbols) for the particle diameter ranging between 0.5 μm and 1.1 μm, whereas the distribution did not change much for particles greater than 1.1 μm. This size-dependent efficiency is typical for depth-type filtration, as used herein^18,19^. In addition, the aging process reduced the number of particles greater than about 1.1 μm in diameter (grey symbols), and the particles less than 1.1 μm mostly remained because heavier particles are likely to deposit at the bottom of the sampling bottle over time^20^ and they are not introduced into the dilution and detection modules (Fig. 1).

Interestingly, a closer look at the concentration data shows a noticeable fluctuation from measurement to measurement (rectangles, triangles, and circles of same-colored symbols in Fig. 2a) relative to the overall average value, as the particle size increases. In particular, the inset of Fig. 2a shows that, for the original fresh slurry, the overall average concentration rapidly decreases from about 100/ml to 25/ml, whereas the fluctuation decreases slowly from about 100/ml to 50/ml with particle diameters ranging between 3 and 13 μm. This feature is commonly observed in the whole range of particle sizes for all slurries. To quantify this fluctuation in the measured data, we define the fluctuation as the relative error *E*, the ratio of the margin of error for 95% confidence interval (CI) to the mean *m*, i.e.,

1 $$\begin{aligned} E \equiv \mathrm {(margin\,of\,error\,for\,95\%\,CI)}/{ m}, \end{aligned}$$

where the margin of error is half the width of 95% CI. Although the measurement of bigger particles can be technically made more accurate than that of smaller particles, the relative error *E* of the three measurements (rectangles, triangles, and circles of same-colored symbols in Fig. 2a) increased and highly fluctuated with particle size, as shown in Fig. 2b.

This seemingly counterintuitive result is a direct consequence of Poisson statistics^15^ in particle counting. Poisson distribution describes the probability of a given number of random events that occur in a constant observation space or time with a fixed average rate of events. Thus, the count of randomly distributed particles in space inherently exhibits Poisson fluctuation, despite all experimental details being precise. In slurry measurement, the dilution process, comprising mixing slurry with deionized water, randomly distributes the large particles across the sample volume; therefore, the measurement of the particle concentration is governed by Poisson statistics. Note that Poisson distribution with an average $\lambda$ has the standard deviation of $\sqrt{\lambda }$^15^, and thus the standard deviation over average increases with decreasing the average. Therefore, the observed fluctuation, i.e., the relative error *E* defined as the margin of error over the mean concentration (Eq. (1)), increases with decreasing particle number count, and seemingly increases with particle diameter (Fig. 2c) because the number of particles decreases with the diameter (Fig. 2a).

## Statistical analysis of inherent fluctuation in the number of large particles

**Figure 3:**
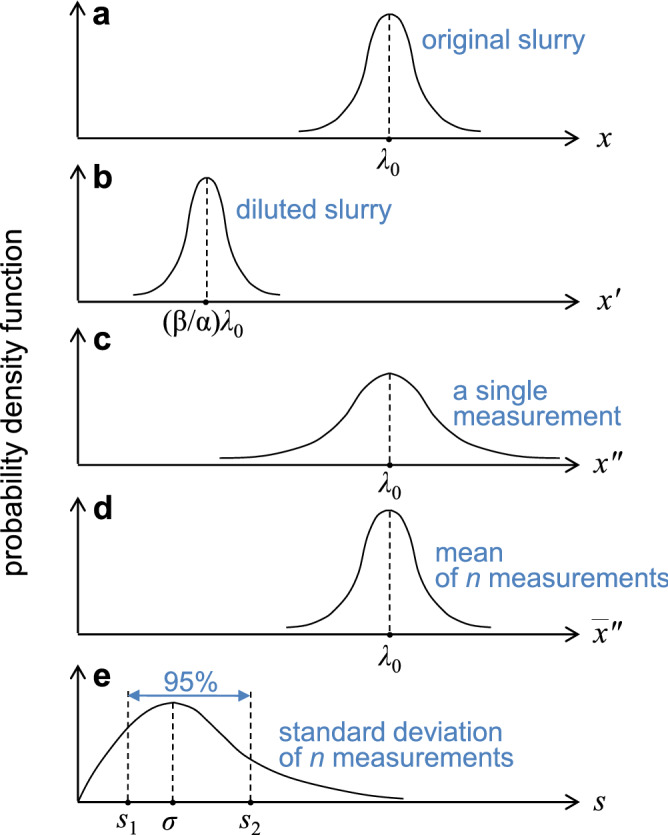
Probability density functions for the number of large particles in the slurry. (**a**) Schematically drawn probability density function for the particle number *x* in 1 *ml* of the original slurry, where the average number of large particles in the original slurry is $\lambda _0$. (**b**) The probability density function for the particle number $x^\prime$ in $\beta$*ml* of the diluted slurry by a factor of $\alpha$. (**c**) The probability density function for the number of particles $x^{\prime \prime }$ in 1 *ml*, obtained by multiplying $\alpha /\beta$ by the measured number $x^\prime$. (**d**) The probability for the mean $\overline{x^{\prime \prime }} = (1/n)\sum _{i=1}^{n} x^{\prime \prime }_i$, where $x^{\prime \prime }_i$ is a random sample from the distribution shown in (**c**). (**e**) The probability density for the standard deviation of n-measurements of $x^{\prime \prime }_i$.

To quantitatively understand the measurement fluctuation, we derive the probability density function (PDF) for the number of particles in 1 ml. Consider that the average number of large particles in 1 *ml* of the original slurry is $\lambda _0$; then, the particle number follows Poisson distribution with average $\lambda _0$ and standard deviation $\sqrt{\lambda _0}$, as schematically described in Fig. 3a. When diluting the slurry by a factor of $\alpha$ and measuring the particle number in $\beta$ ml, the PDF for the particle number $x^\prime$ again follows Poisson distribution with average $(\beta /\alpha )\lambda _0$ and standard deviation $\sqrt{(\beta /\alpha )\lambda _0}$ (Fig. 3b). Further, a Poisson distribution approaches a normal distribution if the average exceeds 10^15^; hence, we approximate the PDF for $x^\prime$ by a normal distribution with the same average and standard deviation. Then, the particle count $x^\prime$ in $\beta$ ml is converted to predict the number of particles $x^{\prime \prime }$ in 1 ml of the original slurry, by multiplying by $\alpha /\beta$. By transforming the random variable $x^\prime$, such as $x^{\prime \prime } =(\alpha /\beta ) x^{\prime }$, we obtain the PDF for $x^{\prime \prime }$ as a normal distribution with average $\lambda _0$ and standard deviation $\sqrt{(\alpha /\beta )\lambda _0}$, as described in Fig. 3c. Experimentally, the measurement is repeated several times, and the mean for the expectation value of the particle number in 1 *ml* is calculated. Let $\overline{x^{\prime \prime }} = (1/n)\sum _{i=1}^{n} x^{\prime \prime }_i$ denote the mean of a random sample $x^{\prime \prime }_i$ of size *n* from the normal distribution for $x^{\prime \prime }$. Then, the PDF of $\overline{x^{\prime \prime }}$ is given by a normal distribution with the mean $\lambda _0$ and standard deviation $\sqrt{(\alpha /(\beta n))\lambda _0}$, as schematically shown in Fig. 3d.

The PDF for the final mean value $\overline{x^{\prime \prime }}$ (Fig. 3d) predicts intrinsic fluctuations, in the order of $\sqrt{(\alpha /(\beta n))\lambda _0}$, in counting the particles in the slurry. Let us consider the 95 % CI, $[ \overline{x^{\prime \prime }}-d, \overline{x^{\prime \prime }}+d ]$ that includes the actual average value $\lambda _0$, i.e., Pr($\overline{x^{\prime \prime }} -d \le \lambda _0 \le \overline{x^{\prime \prime }} + d$) = 0.95, where *d* is the margin of error to be determined. Note that Pr($\overline{x^{\prime \prime }} -d \le \lambda _0 \le \overline{x^{\prime \prime }} + d$) = Pr($-d/(s/\sqrt{n}) \le (\overline{x^{\prime \prime }} - \lambda _0)/(s/\sqrt{n}) \le d/(s/\sqrt{n})$), where *s* is the standard deviation of a random sample of size *n*, i.e., $s=(1/(n-1))\sum _{i=1}^{n} (x^{\prime \prime }_i -\overline{x^{\prime \prime }} )$. As the variable $(\overline{x^{\prime \prime }} - \lambda _0)/(s/\sqrt{n})$ has a *t*-distribution with degrees of freedom $n-1$, we obtain $d=t_{(0.05/2;n-1)}s/\sqrt{n}$. Therefore, the relative error *E* is given by $(t_{(0.05/2;n-1)}s/\sqrt{n})/m$. As a first approximation, we use $s \approx \sigma =\sqrt{(\alpha /(\beta n))\lambda _0}$ and $\lambda _0 \approx m$, which gives

2 $$\begin{aligned}&E =\frac{t_{(0.05/2;n-1)}\frac{s}{\sqrt{n}}}{m} \end{aligned}$$

3 $$\approx t_{(0.05/2;n-1)} \sqrt{\frac{\alpha }{\beta n}}\,m^{-0.5}.$$

Equation (3) shows the inherent fluctuation in the measured value of the mean, which approximately scales with $m^{-0.5}$ with a prefactor accounting for the dilution ratio $\alpha$, measured volume $\beta \,ml$, and the number of measurements *n* in the experiments.

**Figure 4:**
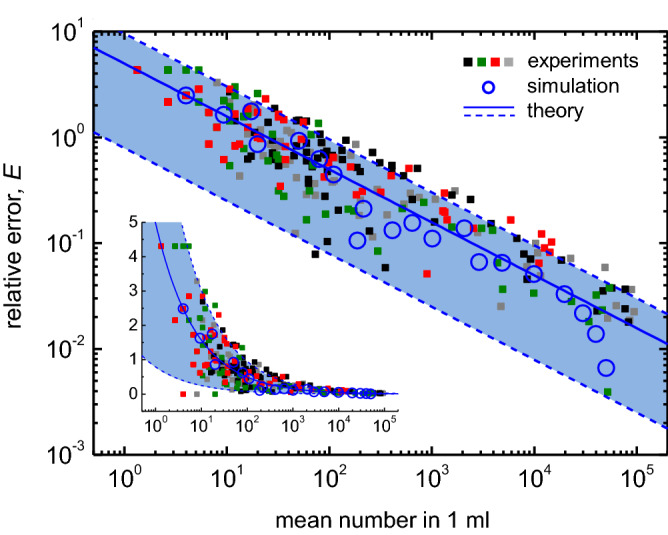
Universal inherent fluctuation in statistical measurement of large particle concentration. Each set of colored dots represents the error defined by Eq. (1), half width of 95% confidence interval divided by the mean, which is obtained from the dataset with the same color shown in Fig. 2. The blue solid curve indicates inherent error, as given in Eq. (3). The dashed blue curves denote the upper and lower bounds of the error, as given in Eqs. (4,5), respectively. The blue empty circles show a Monte Carlo simulation result (see the text for details, section III). The experimental parameters, $\alpha$=60, $\beta$=15, and *n*=3, were used for plotting Eqs. (3) and (4,5). The inset shows the same data plots in log scale on the x-axis.

The range of the standard deviation of *n*-measurements, *s*, provides the upper and lower bounds for *E* (Eq. (2)). Let [$s_1, s_2$] be an interval where *s* lies with 95% confidence, i.e., Pr($s_1 \le s \le s_2$)=0.95. Because Pr($s_1 \le s \le s_2$) = Pr($(n-1)s_1^2/\sigma ^2 \le (n-1)s^2/\sigma ^2 \le (n-1)s_2^2/\sigma ^2$) and the random variable $(n-1)s^2/\sigma ^2$ has a $\chi ^2$-distribution, we obtain $s_1 = \sqrt{\chi ^2_{(0.975;n-1)}{\alpha }/(\beta (n-1))}\,\lambda _0^{+0.5}$ and $s_2 = \sqrt{\chi ^2_{(0.025;n-1)}{\alpha }/(\beta (n-1))}\,\lambda _0^{+0.5}$. The interval [$s_1, s_2$] determines the 95% CI for *E*, as follows.

4 $$E_{\mathrm{low}} = t_{(0.05/2;n-1)}\sqrt{\chi ^2_{(0.975;n-1)}\frac{\alpha }{ (n-1) n \beta }}\,m^{-0.5},$$

5 $$E_{\mathrm{high}} = t_{(0.05/2;n-1)}\sqrt{\chi ^2_{(0.025;n-1)}\frac{\alpha }{ (n-1) n \beta }}\,m^{-0.5},$$

where we have used $\lambda _0 \approx m$. One may derive the exact distribution function for the relative error *E* based on the probability density function of the coefficient of variation^21,22^. However, the empirical approximation, Eqs. (4,5), provide good estimates for the upper and lower bounds of experimentally measured *E* defined by Eq. (1), respectively, and both Eqs. (4,5) converge to Eq. (3) as $n \rightarrow \infty$.

The experimentally measured relative error *E* is in good agreement with the theoretical prediction, Eqs. (4,5) as well as the Monte Carlo simulation, as shown in Fig. 4. Each colored, filled dot (black, green, red, and gray) in Fig. 4 corresponds to the *E* shown in Fig. 2c and is plotted as a function of the measured mean *m*. Overall, the experimentally obtained relative errors excellently scale with $m^{-0.5}$, following the blue solid curve (Eq. (3)), and they mostly lie between the lower and upper bounds indicated by the blue dashed curves (Eqs. (4,5)). A Monte Carlo simulation was performed to simulate particle counts in a given sample volume with different mean numbers, which results in the relative errors, denoted by the blue empty circles in Fig. 4. Remarkably, the upper and lower bounds (Eqs. (4,5)) quantitatively predicts highly fluctuating data at a low mean number, as observed in the experiments (inset of Fig. 4). As the particle number decreases with increasing particle diameter for the slurry investigated herein (Fig. 2a), the seemingly higher *E* at bigger particles (Fig. 2b) is due to the fact that the number of bigger particles is lower than that of smaller particles.

## Statistical control of measurement accuracy

**Figure 5:**
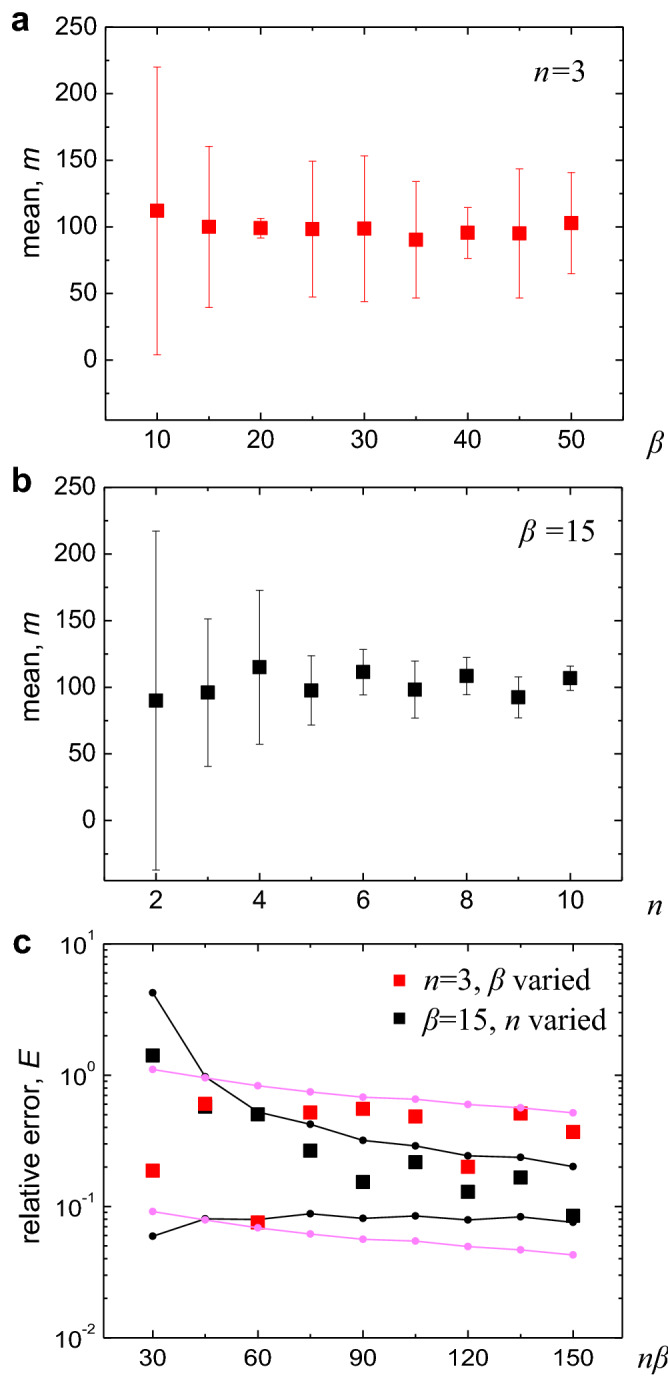
Control of measurement accuracy by varying instrumental parameters, $\beta$**and** *n*. (**a**) The mean value of the particle number in 1 ml with 95$\%$ CI, simulated using a Monte Carlo technique. The particle concentration was obtained with increasing measurement volume $\beta \,ml$, while the number of measurements *n* was maintained constant $n=3$. (**b**) The mean of the particle number in 1 ml with 95$\%$ CI, which was obtained by increasing *n* for a fixed $\beta =15$  ml. (**c**) The relative error, *E*, calculated from the results of (**a**) and (**b**) as a function of the “effective” measurement volume $n \beta$*ml*. For a given $n \beta$ (x-axis), one can obtain the lower value of *E* by increasing *n* rather than by increasing $\beta$. In the simulation, the original undiluted slurry concentration $\lambda _0=100$ in $1 \,ml$ and dilution factor $\alpha =1$ were used.

The measurement accuracy can be enhanced, based on Eqs. (4,5). Practically, one can control the relative error *E* by varying the measurement volume $\beta \,ml$ and the number of measurements *n*, whereas the dilution ratio $\alpha$ is optimized as a specific value and typically fixed owing to the limited resolution in the detection module (Fig. 1). As shown in Fig. 5, the error decreases either by increasing $\beta$ for a given value of $n=3$ (Fig. 5a) or *n* for a given $\beta = 15$ (Fig. 5b). Interestingly, the error *E* rapidly decreases with increasing *n* rather than $\beta$, for the same “effective” measured volume of sample $n \beta \,ml$ (see the x-axis in Fig. 5c). When measuring a high volume of the sample, one can count the high number of particles in the sample and thus lower the relative error *E* by scaling with $\beta ^{-0.5}$ (Eq. (3)). However, one can much efficiently lower the error by increasing *n* rather than $\beta$, where the additional two factors of $t-$ and $\chi ^2 -$distributions associated with the measurement number *n* (Eqs. (4,5) contribute to reducing the error *E*. Thus, for reducing measurement fluctuation, the increase in measurement number *n* is more effective than the increase in the measurement volume factor $\beta$.

The observed fluctuation in the LPC result originates from the statistics of the Poisson distribution associated with instrumental parameters. As the operation parameters of equipment, herein the LPC, often include statistical variables such as the sample volume and the number of measurements, the accuracy of measurement results could depend on the instrumental operation parameters besides the operational accuracy of the instrument. Therefore, one should carefully adjust instrumental parameters to obtain the desired accuracy, particularly when measuring minute volume samples such as a slurry with large particles.

## Conclusion

Our results have practical implications on measuring large particles in slurry solutions. In industry, large particle concentrations must be measured and reported within limited time, and the measurement time is proportional to the measurement volume. Our results (Fig. 5c, Eqs. (4,5)) show that the error *E* can be efficiently reduced by increasing the measurement number *n*, as discussed. Notably, the solution to be measured must be homogeneous. This can be achieved by diluting and mixing the slurry thoroughly (Fig. 1) to ensure that the particles are randomly distributed throughout the sample volume. The randomly distributed particles realize the Poisson distribution of the particle number in a given volume, which is assumed in our theory and simulation. The theoretical and simulation results are in good agreement with the experimental results (Fig. 4), and thus we assume the homogeneity of solutions holds in our system. Still, possible inhomogeneity of the measured solution would lead to the results deviated from our theory.

Our statistics provides the upper and lower bounds of the relative error *E* in the large particle counts. Since the theoretical bounds are based on the 95% confidence interval of the measurements (Fig. 3e), the bounds are expected to include 95% of the data plots in Fig. 4. However, the bounds are shown to include only 87 % of the data. The difference of 8% (= 95% − 87%) originates from both the theoretical limitation and the experimental imperfection. First, the upper and lower bounds of *E* were theoretically derived through the approximation, $mean \approx \lambda _0$ (see Eqs. (4,5) and the text below). Notice that the mean value converges to $\lambda _0$ if the number of measurements $n\rightarrow \infty$, whereas we have used $n=3$ in experiments. This approximation could be partially responsible for the difference of 8% and further can be improved by employing the PDF of the coefficient of variation^21,22^. Second, the instrumental noise and experimental imperfection also attribute the discrepancy 8%. The measuring instrument generally shows technological errors such as electrical and optical noise^23^. Even worse, possible particle aggregation due to the pH-shock during the dilution process (Fig. 1) could alter particle concentration^24^. Also, possible partial inhomogeneity, despite thoroughly mixing and stirring the measured solution, would result in an inaccurate result. This imperfection of experiments, instrumental noise, and unintended chemical reaction could increase the measurement error above the inherent error *E* (Eqs. (4,5)). This indicates that the inherent fluctuation *E* offers the minimum of the error, and we could use the upper and lower bounds of E, Eqs. (4,5), with about 87% confidence level under our experimental conditions. Our results not only provide a fundamental understanding of the inherent fluctuation in counting particles but are also of significance for the practical control of particulate matter in semiconductor manufacturing and related industries.

## Methods

### Sample preparation

We used four slurries for measuring the “large” particle concentration; one original fresh slurry, two filtered slurries, and one slurry obtained by the aging process of the original slurry. The original slurry (black symbol in Fig. 2) is a colloidal silica with a mean particle diameter of 75 nm. The slurry after the aging of three weeks (gray symbol in Fig. 2) shows slightly low large particle counts, compared to the original fresh slurry. Two slurries of different large particle distributions (green and red symbols in Fig. 2) were prepared by filtering the original slurry through two depth-type filters with nominal pore sizes of 0.7 and 0.1 μm, respectively (green symbols by 0.7 μm, red by 0.1 μm).

### Measurement of “Large” particles in the slurry

We used AccuSizer 780 APS (Particle Sizing Systems, Santa Barbara, California, USA) to measure the large particles, which employs the light scattering technique and detects particles of diameter ≥ 0.5 μm. The dilution factor of $\alpha =60$ and the measurement volume factor of $\beta =15$, i.e., 15 ml were used for all experiments. The flow rate was 15 ml/min.

### Monte-Carlo simulation

**Figure 6:**
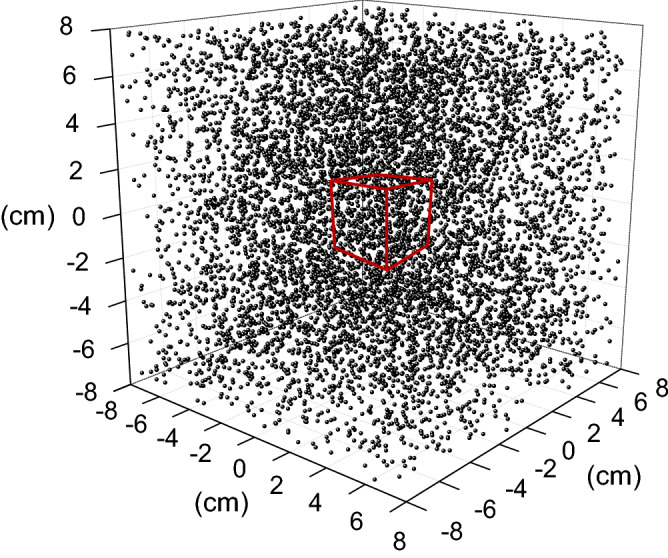
Snapshot of the randomly distributed particles and measurement volume. The total number of particles was 7,683, which were distributed throughout the entire volume of the simulation space, i.e., across 3,824 ml, representing a diluted solution by a factor of 60 with an original concentration of 120/ml. The red box indicates the observation volume of 30 ml.

A Monte-Carlo simulation was performed to investigate the statistics of particle counting in the slurry. We first randomly distributed the particles in a given volume of slurry, and counted the particles that reside within an observation volume. We used a software “Mathematica” for the simulation with parameters relevant to the experiments. For example, the snapshot below represents a solution diluted by a factor of $\alpha =60$, the resulting concentration of 2/ml, and the observation volume of 30 ml, indicated by red box (Fig. 6).
